# Aqua­bis­[4-(methyl­sulfan­yl)benzoato-κ*O*]bis­(pyridine-κ*N*)copper(II)

**DOI:** 10.1107/S1600536810035336

**Published:** 2010-09-08

**Authors:** Guo-Qing Jiang, Jian-Hua Chen, Miao Wang, Yu-Jun Shi

**Affiliations:** a College of Chemistry and Chemical Engineering, Nantong University, Nantong 226009, People’s Republic of China

## Abstract

In the title mol­ecule, [Cu(C_8_H_7_O_2_S)_2_(C_5_H_5_N)_2_(H_2_O)], the Cu^II^ ion is penta­coordinated in a distorted square-pyramidal geometry by two O atoms of two 4-(methyl­sulfan­yl)benzoate anions and two N atoms of two pyridine ligands and a water O atom situated at the apical site. In the crystal structure, O—H⋯O hydrogen bonds link mol­ecules into chains along the *b* axis.

## Related literature

For the pharmacological properties of thio­amino acid for treating copper intoxication, see: Tran-Ho *et al.* (1997 ▶). For the catalytic properties of copper(II) complexes, see: Kawasaki & Katsuki (1997 ▶).
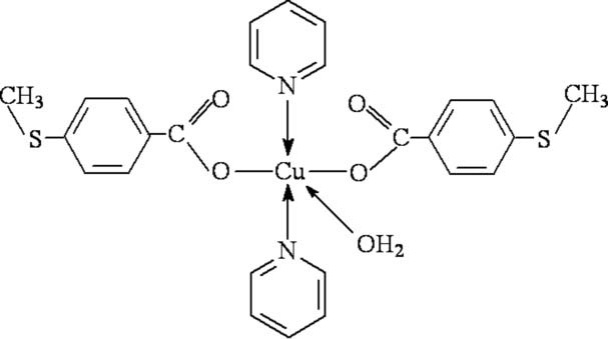


## Experimental

### 

#### Crystal data

[Cu(C_8_H_7_O_2_S)_2_(C_5_H_5_N)_2_(H_2_O)]
*M* *_r_* = 574.15
Monoclinic, 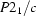

*a* = 25.676 (5) Å
*b* = 6.0030 (11) Å
*c* = 17.026 (3) Å
β = 97.753 (4)°
*V* = 2600.3 (8) Å^3^
*Z* = 4
Mo *K*α radiation
μ = 1.04 mm^−1^
*T* = 293 K
0.22 × 0.16 × 0.10 mm

#### Data collection

Bruker SMART APEX CCD area-detector diffractometer
Absorption correction: multi-scan (*SADABS*; Bruker, 2000 ▶) *T*_min_ = 0.885, *T*_max_ = 0.903
14401 measured reflections
4822 independent reflections
3640 reflections with *I* > 2σ(*I*)
*R*_int_ = 0.034

#### Refinement

*R*[*F* ^2^ > 2σ(*F* ^2^)] = 0.035
*wR*(*F* ^2^) = 0.106
*S* = 1.00
4822 reflections
332 parameters
20 restraints
H atoms treated by a mixture of independent and constrained refinement
Δρ_max_ = 0.28 e Å^−3^
Δρ_min_ = −0.32 e Å^−3^

### 

Data collection: *SMART* (Bruker, 2000 ▶); cell refinement: *SAINT* (Bruker, 2000 ▶); data reduction: *SAINT*; program(s) used to solve structure: *SHELXTL* (Sheldrick, 2008 ▶); program(s) used to refine structure: *SHELXTL*; molecular graphics: *SHELXTL*; software used to prepare material for publication: *SHELXTL*.

## Supplementary Material

Crystal structure: contains datablocks global, I. DOI: 10.1107/S1600536810035336/cv2757sup1.cif

Structure factors: contains datablocks I. DOI: 10.1107/S1600536810035336/cv2757Isup2.hkl

Additional supplementary materials:  crystallographic information; 3D view; checkCIF report

## Figures and Tables

**Table 1:** Hydrogen-bond geometry (Å, °)

*D*—H⋯*A*	*D*—H	H⋯*A*	*D*⋯*A*	*D*—H⋯*A*
O1*W*—H1*WA*⋯O3^i^	0.83 (2)	1.88 (2)	2.701 (3)	170 (3)
O1*W*—H1*WB*⋯O1^i^	0.83 (2)	1.89 (2)	2.721 (3)	173 (3)

Symmetry code: (i) 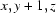.

**Fig. 1.**
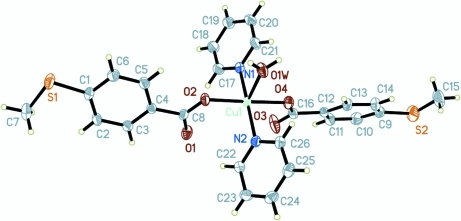
View of (I) with the atomic numbering scheme. Displacement ellipsoids are drawn at the 30% probability level.

**Fig. 2.**
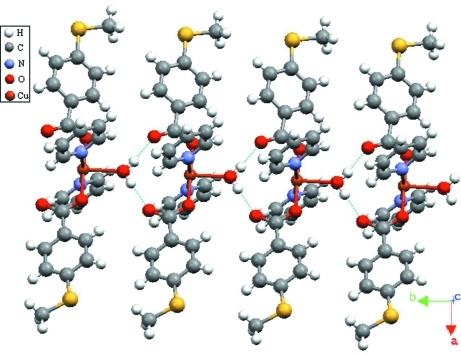
A portion of the crystal packing showing a hydrogen-bonded (dashed lones) chain.

## supplementary crystallographic information

### Comment

The design and synthesis of metal-organic complexes have attracted intense attention in recent years owing to their potential practical applications, such as biological activities, magnetism and catalysis (for the catalytic properties of copper (II) complexes, see Kawasaki *et al.* (1997)). Also the pharmacological properties of thioamino acid derivatives for treating copper intoxication are well-known (Tran-Ho *et al.*, 1997). In order to achieve supramolecular transition metal complexes by self-assembly, and to explore the relationship between the structure and the biological properties, as one part of our systematic work, in this paper, we report on the synthesis and crystal structure of the title compound, (I) (Fig. 1).

In (I), the Cu^II^ ion has a square- pyramidal environment being coordinated by two carboxylate O atoms from the two coordinating 4-(methylsulfanyl)benzoate ligands and two N atoms from the two pyridine ligands, and an O atom from the water molecule. A long apical Cu—OH_2_ bond of 2.243 (2) Å is due to the Jahn-Teller effect. In the crystal structure, intermolecular O—H···O hydrogen bonds (Table 1) link the molecules translated along axis *b* into chains (Fig. 2).

### Experimental

4-(methylsulfanyl) benzoic acid (0.35 g, 2 mmol) was dissolved in pydine (5 ml) and then Cu(NO_3_)_2_^.^3H_2_O (0.240 g, 1 mmol) was added. After refluxing for 2 h, the solvent was removed *in vacuo*. The resulting mixture was dissolved in CH_2_Cl_2_ and single crystals were obtained by diffusion ethyl ether into the filtrate.

### Refinement

Carbon-bound H atoms were positioned geometrically, with C—H = 0.96Å for methyl groups and 0.93 Å for aromatic rings, and refined using a riding model, with *U*_iso_ (H) = 1.2-1.5 *U*_eq_ (C). The water' H atoms were located on a difference map and isotropically refined.

### Crystal data

**Table:**

[Cu(C_8_H_7_O_2_S)_2_(C_5_H_5_N)_2_(H_2_O)]	*F*(000) = 1188
*M**_r_* = 574.15	*D*_x_ = 1.467 Mg m^−^^3^
Monoclinic, *P*2_1_/*c*	Mo *K*α radiation, λ = 0.71073 Å
Hall symbol: -P 2ybc	Cell parameters from 4039 reflections
*a* = 25.676 (5) Å	θ = 2.4–27.2°
*b* = 6.0030 (11) Å	µ = 1.04 mm^−^^1^
*c* = 17.026 (3) Å	*T* = 293 K
β = 97.753 (4)°	Prism, blue
*V* = 2600.3 (8) Å^3^	0.22 × 0.16 × 0.10 mm
*Z* = 4	

### Data collection

**Table:**

Bruker SMART APEX CCD area-detector diffractometer	4822 independent reflections
Radiation source: fine-focus sealed tube	3640 reflections with *I* > 2σ(*I*)
graphite	*R*_int_ = 0.034
phi and ω scans	θ_max_ = 25.5°, θ_min_ = 1.6°
Absorption correction: multi-scan (*SADABS*; Bruker, 2000)	*h* = −30→31
*T*_min_ = 0.885, *T*_max_ = 0.903	*k* = −5→7
14401 measured reflections	*l* = −20→20

### Refinement

**Table:**

Refinement on *F*^2^	Primary atom site location: structure-invariant direct methods
Least-squares matrix: full	Secondary atom site location: difference Fourier map
*R*[*F*^2^ > 2σ(*F*^2^)] = 0.035	Hydrogen site location: inferred from neighbouring sites
*wR*(*F*^2^) = 0.106	H atoms treated by a mixture of independent and constrained refinement
*S* = 1.00	*w* = 1/[σ^2^(*F*_o_^2^) + (0.0646*P*)^2^] where *P* = (*F*_o_^2^ + 2*F*_c_^2^)/3
4822 reflections	(Δ/σ)_max_ = 0.003
332 parameters	Δρ_max_ = 0.28 e Å^−^^3^
20 restraints	Δρ_min_ = −0.32 e Å^−^^3^

### Special details

**Table:**

Geometry. All e.s.d.'s (except the e.s.d. in the dihedral angle between two l.s. planes) are estimated using the full covariance matrix. The cell e.s.d.'s are taken into account individually in the estimation of e.s.d.'s in distances, angles and torsion angles; correlations between e.s.d.'s in cell parameters are only used when they are defined by crystal symmetry. An approximate (isotropic) treatment of cell e.s.d.'s is used for estimating e.s.d.'s involving l.s. planes.
Refinement. Refinement of *F*^2^ against ALL reflections. The weighted *R*-factor *wR* and goodness of fit *S* are based on *F*^2^, conventional *R*-factors *R* are based on *F*, with *F* set to zero for negative *F*^2^. The threshold expression of *F*^2^ > σ(*F*^2^) is used only for calculating *R*-factors(gt) *etc*. and is not relevant to the choice of reflections for refinement. *R*-factors based on *F*^2^ are statistically about twice as large as those based on *F*, and *R*- factors based on ALL data will be even larger.

### Fractional atomic coordinates and isotropic or equivalent isotropic displacement parameters (Å^2^)

**Table:**

	*x*	*y*	*z*	*U*_iso_*/*U*_eq_	
Cu1	0.743521 (11)	0.02678 (5)	0.137765 (17)	0.03387 (12)	
S1	0.45561 (3)	−0.03164 (14)	−0.19282 (5)	0.0620 (2)	
S2	1.05166 (4)	−0.0851 (2)	0.41678 (7)	0.0901 (4)	
C1	0.50925 (10)	−0.0745 (5)	−0.11836 (15)	0.0409 (6)	
C2	0.51918 (10)	−0.2719 (5)	−0.07666 (17)	0.0464 (7)	
H2	0.4961	−0.3912	−0.0864	0.056*	
C3	0.56350 (10)	−0.2922 (4)	−0.02033 (15)	0.0415 (6)	
H3	0.5699	−0.4262	0.0066	0.050*	
C4	0.59820 (9)	−0.1168 (4)	−0.00363 (14)	0.0341 (5)	
C5	0.58709 (10)	0.0812 (5)	−0.04404 (16)	0.0434 (6)	
H5	0.6094	0.2023	−0.0327	0.052*	
C6	0.54381 (11)	0.1033 (5)	−0.10060 (16)	0.0475 (7)	
H6	0.5375	0.2379	−0.1272	0.057*	
C7	0.41908 (12)	−0.2863 (6)	−0.1935 (2)	0.0723 (10)	
H7A	0.3887	−0.2776	−0.2329	0.108*	
H7B	0.4082	−0.3094	−0.1423	0.108*	
H7C	0.4408	−0.4084	−0.2056	0.108*	
C8	0.64799 (9)	−0.1484 (5)	0.05272 (14)	0.0371 (6)	
C9	0.98963 (11)	−0.0843 (5)	0.35707 (17)	0.0519 (7)	
C10	0.97517 (11)	−0.2785 (5)	0.31685 (18)	0.0564 (8)	
H10	0.9984	−0.3980	0.3200	0.068*	
C11	0.92676 (10)	−0.2969 (5)	0.27216 (17)	0.0493 (7)	
H11	0.9171	−0.4307	0.2468	0.059*	
C12	0.89200 (9)	−0.1183 (5)	0.26422 (15)	0.0378 (6)	
C13	0.90713 (11)	0.0767 (5)	0.30264 (16)	0.0447 (6)	
H13	0.8845	0.1984	0.2976	0.054*	
C14	0.95556 (11)	0.0948 (5)	0.34880 (17)	0.0539 (8)	
H14	0.9652	0.2283	0.3744	0.065*	
C15	1.06228 (15)	0.1976 (8)	0.4476 (3)	0.1061 (16)	
H15A	1.0967	0.2120	0.4772	0.159*	
H15B	1.0363	0.2403	0.4803	0.159*	
H15C	1.0596	0.2924	0.4018	0.159*	
C16	0.83848 (10)	−0.1431 (5)	0.21525 (16)	0.0440 (6)	
C17	0.77130 (11)	−0.1691 (5)	−0.01263 (17)	0.0519 (6)	
H17A	0.7478	−0.2801	−0.0025	0.062*	
C18	0.79446 (12)	−0.1837 (5)	−0.08029 (17)	0.0555 (6)	
H18A	0.7870	−0.3029	−0.1148	0.067*	
C19	0.82878 (12)	−0.0210 (5)	−0.09659 (18)	0.0556 (6)	
H19A	0.8443	−0.0257	−0.1428	0.067*	
C20	0.83972 (12)	0.1497 (6)	−0.04289 (17)	0.0590 (8)	
H20	0.8634	0.2614	−0.0517	0.071*	
C21	0.81514 (11)	0.1524 (5)	0.02388 (17)	0.0533 (8)	
H21	0.8228	0.2682	0.0599	0.064*	
C22	0.67778 (10)	−0.1574 (5)	0.25511 (15)	0.0442 (6)	
H22	0.6689	−0.2670	0.2171	0.053*	
C23	0.65702 (11)	−0.1699 (5)	0.32568 (17)	0.0507 (7)	
H23	0.6349	−0.2870	0.3349	0.061*	
C24	0.66933 (12)	−0.0074 (5)	0.38224 (17)	0.0513 (7)	
H24	0.6559	−0.0130	0.4302	0.062*	
C25	0.70184 (12)	0.1627 (5)	0.36627 (17)	0.0535 (8)	
H25	0.7105	0.2757	0.4031	0.064*	
C26	0.72176 (11)	0.1645 (5)	0.29452 (15)	0.0462 (7)	
H26	0.7440	0.2799	0.2842	0.055*	
N1	0.78083 (8)	−0.0024 (3)	0.03984 (13)	0.0399 (5)	
N2	0.71013 (8)	0.0062 (3)	0.23945 (12)	0.0381 (5)	
O1	0.66052 (7)	−0.3399 (3)	0.07546 (11)	0.0523 (5)	
O2	0.67505 (7)	0.0256 (3)	0.07148 (11)	0.0448 (5)	
O3	0.82353 (9)	−0.3270 (4)	0.19108 (17)	0.0859 (8)	
O4	0.81221 (7)	0.0363 (3)	0.20312 (11)	0.0444 (5)	
O1W	0.74339 (8)	0.4003 (4)	0.13501 (13)	0.0558 (6)	
H1WA	0.7665 (10)	0.495 (4)	0.148 (2)	0.067*	
H1WB	0.7188 (10)	0.478 (5)	0.1131 (18)	0.067*	

### Atomic displacement parameters (Å^2^)

**Table:**

	*U*^11^	*U*^22^	*U*^33^	*U*^12^	*U*^13^	*U*^23^
Cu1	0.02653 (17)	0.0401 (2)	0.03353 (19)	−0.00232 (12)	−0.00137 (12)	0.00054 (13)
S1	0.0502 (5)	0.0705 (6)	0.0579 (5)	0.0065 (4)	−0.0202 (4)	0.0042 (4)
S2	0.0474 (5)	0.1133 (9)	0.0982 (8)	−0.0033 (5)	−0.0316 (5)	0.0246 (6)
C1	0.0335 (13)	0.0487 (17)	0.0383 (14)	0.0056 (12)	−0.0030 (11)	−0.0035 (12)
C2	0.0362 (14)	0.0432 (16)	0.0563 (17)	−0.0065 (12)	−0.0066 (12)	−0.0001 (13)
C3	0.0377 (14)	0.0403 (15)	0.0449 (15)	−0.0017 (12)	−0.0007 (11)	0.0066 (12)
C4	0.0277 (12)	0.0388 (15)	0.0357 (13)	0.0005 (11)	0.0033 (10)	−0.0010 (11)
C5	0.0412 (15)	0.0377 (16)	0.0491 (16)	−0.0042 (12)	−0.0020 (12)	−0.0006 (12)
C6	0.0491 (17)	0.0372 (16)	0.0541 (17)	0.0052 (13)	−0.0004 (13)	0.0070 (12)
C7	0.0495 (18)	0.091 (3)	0.070 (2)	−0.0060 (18)	−0.0178 (16)	−0.0078 (18)
C8	0.0288 (12)	0.0525 (18)	0.0295 (13)	0.0012 (12)	0.0020 (10)	−0.0004 (11)
C9	0.0363 (15)	0.068 (2)	0.0481 (17)	−0.0031 (14)	−0.0054 (12)	0.0142 (15)
C10	0.0419 (16)	0.060 (2)	0.065 (2)	0.0138 (14)	0.0010 (14)	0.0139 (16)
C11	0.0425 (15)	0.0414 (17)	0.0617 (19)	0.0044 (13)	−0.0017 (13)	0.0033 (13)
C12	0.0308 (13)	0.0425 (15)	0.0397 (14)	−0.0015 (11)	0.0029 (11)	0.0050 (11)
C13	0.0395 (15)	0.0477 (17)	0.0451 (16)	0.0044 (13)	−0.0012 (12)	−0.0029 (12)
C14	0.0459 (17)	0.058 (2)	0.0548 (18)	−0.0042 (14)	−0.0053 (14)	−0.0064 (14)
C15	0.069 (3)	0.121 (4)	0.116 (4)	−0.032 (3)	−0.032 (2)	0.003 (3)
C16	0.0338 (14)	0.0492 (18)	0.0476 (16)	−0.0039 (13)	−0.0003 (12)	0.0055 (13)
C17	0.0516 (13)	0.0577 (15)	0.0475 (13)	−0.0102 (11)	0.0108 (11)	−0.0042 (11)
C18	0.0569 (13)	0.0613 (15)	0.0499 (13)	−0.0086 (11)	0.0129 (11)	−0.0094 (11)
C19	0.0542 (13)	0.0644 (15)	0.0506 (13)	−0.0081 (11)	0.0157 (11)	−0.0053 (11)
C20	0.0530 (18)	0.074 (2)	0.0526 (18)	−0.0221 (16)	0.0170 (15)	−0.0016 (15)
C21	0.0448 (16)	0.060 (2)	0.0546 (18)	−0.0162 (14)	0.0063 (14)	−0.0073 (14)
C22	0.0423 (15)	0.0474 (17)	0.0421 (15)	−0.0053 (13)	0.0028 (12)	0.0003 (12)
C23	0.0491 (17)	0.0527 (19)	0.0514 (18)	−0.0027 (14)	0.0108 (14)	0.0084 (14)
C24	0.0525 (17)	0.063 (2)	0.0395 (15)	0.0116 (15)	0.0114 (13)	0.0025 (13)
C25	0.0577 (18)	0.058 (2)	0.0436 (17)	0.0038 (15)	0.0017 (14)	−0.0129 (13)
C26	0.0455 (16)	0.0506 (18)	0.0409 (16)	−0.0058 (13)	0.0000 (12)	−0.0047 (12)
N1	0.0318 (11)	0.0475 (14)	0.0391 (12)	−0.0038 (9)	−0.0001 (9)	0.0010 (9)
N2	0.0329 (11)	0.0453 (14)	0.0348 (11)	−0.0004 (9)	0.0003 (9)	−0.0005 (9)
O1	0.0463 (11)	0.0509 (13)	0.0545 (12)	0.0128 (9)	−0.0120 (9)	0.0022 (9)
O2	0.0319 (9)	0.0566 (12)	0.0432 (11)	−0.0077 (8)	−0.0049 (8)	−0.0044 (8)
O3	0.0617 (15)	0.0459 (15)	0.136 (2)	−0.0108 (11)	−0.0391 (15)	−0.0075 (13)
O4	0.0307 (9)	0.0524 (12)	0.0477 (11)	0.0057 (8)	−0.0039 (8)	−0.0022 (8)
O1W	0.0399 (11)	0.0351 (12)	0.0856 (16)	−0.0016 (8)	−0.0157 (11)	0.0048 (10)

### Geometric parameters (Å, °)

**Table:**

Cu1—O4	1.9550 (18)	C13—C14	1.383 (4)
Cu1—O2	1.9567 (18)	C13—H13	0.9300
Cu1—N2	2.037 (2)	C14—H14	0.9300
Cu1—N1	2.039 (2)	C15—H15A	0.9600
Cu1—O1W	2.243 (2)	C15—H15B	0.9600
S1—C1	1.761 (3)	C15—H15C	0.9600
S1—C7	1.793 (4)	C16—O3	1.222 (3)
S2—C9	1.770 (3)	C16—O4	1.272 (3)
S2—C15	1.786 (5)	C17—N1	1.342 (3)
C1—C2	1.387 (4)	C17—C18	1.369 (4)
C1—C6	1.395 (4)	C17—H17A	0.9300
C2—C3	1.391 (3)	C18—C19	1.368 (4)
C2—H2	0.9300	C18—H18A	0.9300
C3—C4	1.384 (3)	C19—C20	1.377 (4)
C3—H3	0.9300	C19—H19A	0.9300
C4—C5	1.384 (4)	C20—C21	1.373 (4)
C4—C8	1.503 (3)	C20—H20	0.9300
C5—C6	1.375 (4)	C21—N1	1.333 (3)
C5—H5	0.9300	C21—H21	0.9300
C6—H6	0.9300	C22—N2	1.336 (3)
C7—H7A	0.9600	C22—C23	1.381 (4)
C7—H7B	0.9600	C22—H22	0.9300
C7—H7C	0.9600	C23—C24	1.377 (4)
C8—O1	1.241 (3)	C23—H23	0.9300
C8—O2	1.271 (3)	C24—C25	1.369 (4)
C9—C10	1.377 (4)	C24—H24	0.9300
C9—C14	1.381 (4)	C25—C26	1.387 (4)
C10—C11	1.371 (4)	C25—H25	0.9300
C10—H10	0.9300	C26—N2	1.340 (3)
C11—C12	1.390 (4)	C26—H26	0.9300
C11—H11	0.9300	O1W—H1WA	0.826 (18)
C12—C13	1.371 (4)	O1W—H1WB	0.832 (18)
C12—C16	1.515 (3)		
			
O4—Cu1—O2	178.45 (8)	C9—C14—C13	120.3 (3)
O4—Cu1—N2	88.21 (8)	C9—C14—H14	119.8
O2—Cu1—N2	92.39 (8)	C13—C14—H14	119.8
O4—Cu1—N1	88.89 (8)	S2—C15—H15A	109.5
O2—Cu1—N1	90.73 (8)	S2—C15—H15B	109.5
N2—Cu1—N1	171.03 (8)	H15A—C15—H15B	109.5
O4—Cu1—O1W	88.93 (7)	S2—C15—H15C	109.5
O2—Cu1—O1W	89.59 (7)	H15A—C15—H15C	109.5
N2—Cu1—O1W	94.51 (8)	H15B—C15—H15C	109.5
N1—Cu1—O1W	93.91 (8)	O3—C16—O4	125.0 (3)
C1—S1—C7	103.76 (14)	O3—C16—C12	119.6 (3)
C9—S2—C15	104.84 (17)	O4—C16—C12	115.3 (2)
C2—C1—C6	118.4 (2)	N1—C17—C18	123.2 (3)
C2—C1—S1	124.4 (2)	N1—C17—H17A	118.4
C6—C1—S1	117.2 (2)	C18—C17—H17A	118.4
C1—C2—C3	120.3 (2)	C17—C18—C19	119.3 (3)
C1—C2—H2	119.9	C17—C18—H18A	120.3
C3—C2—H2	119.9	C19—C18—H18A	120.3
C4—C3—C2	121.3 (2)	C18—C19—C20	118.4 (3)
C4—C3—H3	119.4	C18—C19—H19A	120.8
C2—C3—H3	119.4	C20—C19—H19A	120.8
C3—C4—C5	117.9 (2)	C21—C20—C19	118.9 (3)
C3—C4—C8	120.2 (2)	C21—C20—H20	120.6
C5—C4—C8	121.8 (2)	C19—C20—H20	120.6
C6—C5—C4	121.6 (3)	N1—C21—C20	123.4 (3)
C6—C5—H5	119.2	N1—C21—H21	118.3
C4—C5—H5	119.2	C20—C21—H21	118.3
C5—C6—C1	120.6 (3)	N2—C22—C23	122.5 (3)
C5—C6—H6	119.7	N2—C22—H22	118.8
C1—C6—H6	119.7	C23—C22—H22	118.8
S1—C7—H7A	109.5	C24—C23—C22	119.4 (3)
S1—C7—H7B	109.5	C24—C23—H23	120.3
H7A—C7—H7B	109.5	C22—C23—H23	120.3
S1—C7—H7C	109.5	C25—C24—C23	118.5 (3)
H7A—C7—H7C	109.5	C25—C24—H24	120.7
H7B—C7—H7C	109.5	C23—C24—H24	120.7
O1—C8—O2	124.9 (2)	C24—C25—C26	119.2 (3)
O1—C8—C4	118.6 (2)	C24—C25—H25	120.4
O2—C8—C4	116.4 (2)	C26—C25—H25	120.4
C10—C9—C14	118.9 (3)	N2—C26—C25	122.5 (3)
C10—C9—S2	116.4 (2)	N2—C26—H26	118.8
C14—C9—S2	124.7 (3)	C25—C26—H26	118.8
C11—C10—C9	120.5 (3)	C21—N1—C17	116.7 (2)
C11—C10—H10	119.8	C21—N1—Cu1	120.20 (19)
C9—C10—H10	119.8	C17—N1—Cu1	123.01 (18)
C10—C11—C12	120.9 (3)	C22—N2—C26	117.9 (2)
C10—C11—H11	119.5	C22—N2—Cu1	123.94 (18)
C12—C11—H11	119.5	C26—N2—Cu1	118.18 (18)
C13—C12—C11	118.4 (2)	C8—O2—Cu1	124.47 (17)
C13—C12—C16	121.6 (2)	C16—O4—Cu1	119.04 (17)
C11—C12—C16	120.0 (2)	Cu1—O1W—H1WA	133 (2)
C12—C13—C14	120.9 (3)	Cu1—O1W—H1WB	125 (2)
C12—C13—H13	119.5	H1WA—O1W—H1WB	102 (3)
C14—C13—H13	119.5		
			
C7—S1—C1—C2	3.5 (3)	C23—C24—C25—C26	−0.8 (4)
C7—S1—C1—C6	−176.4 (2)	C24—C25—C26—N2	0.5 (4)
C6—C1—C2—C3	−1.9 (4)	C20—C21—N1—C17	1.1 (4)
S1—C1—C2—C3	178.1 (2)	C20—C21—N1—Cu1	−176.6 (2)
C1—C2—C3—C4	0.9 (4)	C18—C17—N1—C21	−0.7 (4)
C2—C3—C4—C5	0.9 (4)	C18—C17—N1—Cu1	176.9 (2)
C2—C3—C4—C8	−175.2 (2)	O4—Cu1—N1—C21	−57.5 (2)
C3—C4—C5—C6	−1.7 (4)	O2—Cu1—N1—C21	121.0 (2)
C8—C4—C5—C6	174.3 (2)	N2—Cu1—N1—C21	−128.6 (5)
C4—C5—C6—C1	0.7 (4)	O1W—Cu1—N1—C21	31.4 (2)
C2—C1—C6—C5	1.1 (4)	O4—Cu1—N1—C17	125.0 (2)
S1—C1—C6—C5	−178.9 (2)	O2—Cu1—N1—C17	−56.5 (2)
C3—C4—C8—O1	9.3 (4)	N2—Cu1—N1—C17	53.8 (6)
C5—C4—C8—O1	−166.5 (2)	O1W—Cu1—N1—C17	−146.2 (2)
C3—C4—C8—O2	−172.8 (2)	C23—C22—N2—C26	−1.0 (4)
C5—C4—C8—O2	11.3 (3)	C23—C22—N2—Cu1	178.0 (2)
C15—S2—C9—C10	171.5 (3)	C25—C26—N2—C22	0.4 (4)
C15—S2—C9—C14	−8.7 (3)	C25—C26—N2—Cu1	−178.7 (2)
C14—C9—C10—C11	−2.9 (5)	O4—Cu1—N2—C22	−123.9 (2)
S2—C9—C10—C11	176.9 (2)	O2—Cu1—N2—C22	57.5 (2)
C9—C10—C11—C12	2.2 (4)	N1—Cu1—N2—C22	−52.7 (6)
C10—C11—C12—C13	−0.4 (4)	O1W—Cu1—N2—C22	147.3 (2)
C10—C11—C12—C16	−179.4 (3)	O4—Cu1—N2—C26	55.2 (2)
C11—C12—C13—C14	−0.6 (4)	O2—Cu1—N2—C26	−123.38 (19)
C16—C12—C13—C14	178.3 (2)	N1—Cu1—N2—C26	126.4 (5)
C10—C9—C14—C13	1.8 (4)	O1W—Cu1—N2—C26	−33.6 (2)
S2—C9—C14—C13	−177.9 (2)	O1—C8—O2—Cu1	2.0 (4)
C12—C13—C14—C9	−0.1 (4)	C4—C8—O2—Cu1	−175.66 (15)
C13—C12—C16—O3	−169.9 (3)	O4—Cu1—O2—C8	167 (3)
C11—C12—C16—O3	9.0 (4)	N2—Cu1—O2—C8	−80.3 (2)
C13—C12—C16—O4	9.7 (4)	N1—Cu1—O2—C8	91.3 (2)
C11—C12—C16—O4	−171.4 (2)	O1W—Cu1—O2—C8	−174.8 (2)
N1—C17—C18—C19	−0.6 (5)	O3—C16—O4—Cu1	−2.5 (4)
C17—C18—C19—C20	1.5 (5)	C12—C16—O4—Cu1	178.00 (16)
C18—C19—C20—C21	−1.2 (5)	O2—Cu1—O4—C16	−151 (3)
C19—C20—C21—N1	−0.1 (5)	N2—Cu1—O4—C16	96.6 (2)
N2—C22—C23—C24	0.7 (4)	N1—Cu1—O4—C16	−74.9 (2)
C22—C23—C24—C25	0.2 (4)	O1W—Cu1—O4—C16	−168.8 (2)

### Hydrogen-bond geometry (Å, °)

**Table:**

*D*—H···*A*	*D*—H	H···*A*	*D*···*A*	*D*—H···*A*
O1W—H1WA···O3^i^	0.83 (2)	1.88 (2)	2.701 (3)	170 (3)
O1W—H1WB···O1^i^	0.83 (2)	1.89 (2)	2.721 (3)	173 (3)

Symmetry codes: (i) *x*, *y*+1, *z*.
